# Burnout syndrome among pediatric dentists in Egypt

**DOI:** 10.1186/s43045-022-00230-z

**Published:** 2022-09-21

**Authors:** Mohamed Zayed Radwan, Mahmoud Morsy

**Affiliations:** 1 Pediatric Dentistry, Ain Shams University, Cairo, Egypt; 2 Psychiatry, Ain Shams University, Cairo, Egypt

**Keywords:** Burnout, Pediatric dentists, Depersonalization, Emotional exhaustion, Personal accomplishment

## Abstract

**Background:**

Pediatric dentists in Egypt are exposed to chronic stress associated with pediatric dental care. After long years of specialization, training, and practice, they seem to be unsatisfied. An increased prevalence of burnout could be the reason. This study aimed to determine the prevalence of occupational burnout among Egyptian pediatric dentists.

This study is a cross-sectional, observational study, which was carried by a self-administered online survey questionnaire that was sent to active members in the Egyptian Pediatric Dentistry Associations. A total number of 100 pediatric dentists participated in this study. All of them were offered to fulfill the questionnaires including that of Maslach Burnout Inventory and the semi-structured questionnaire to complete their sociodemographic and work-related data.

**Results:**

It was found that the number of kids raised up by the practitioner is significantly associated with the level of depersonalization. It was also found that the number of working hours per week and number of patients treated per day have a significant association with the level of exhaustion, depersonalization, and personal accomplishment. There were no gender differences in the prevalence of burnout or depression.

**Conclusions:**

Burnout prevalence among pediatric dentist in Egypt is high and higher than its prevalence among other dentists. Marriage and sleeping in home with the family have protective effects against burnout statistically. There is significant association between high number of working hours of the pediatric dentist per week and burnout.

## Background

### *Professional burnout*

Herbert Freudenberger was a pioneer in describing occupational burnout as a set of symptoms such as physical fatigue, emotional exhaustion, and loss of motivation which are connected to a job negative psychological state. He also pointed out signs of burnout, for instance, losing connection with family and friends or ignoring personal commitment and hobbies for work reasons [1].

“Health care professionals should be committed, involved, dedicated, and concerned, but not at the cost of their entire lives outside work” as said by Freundenberger highlighting the effect of healthcare careers on an individual. Frustration, anger, and burnout can result from working constantly. Freunderberger defined burnout as “the extinction of motivation or incentive, especially where one's devotion to a cause or relationship fails to produce the desired results” [2–4].

Maslach et al. described burnout in terms of three dimensions: emotional exhaustion, depersonalization, and reduced personal accomplishment. It occurs more often in individuals who serve people directly. Depersonalization includes cynical and negative attitudes and feelings towards one’s patients and clients, which can lead to a “dehumanized” view of one’s patients or clients. Depersonalization and emotional exhaustion are related. Reduced personal accomplishment includes thoughts and attitudes related to lack of professional satisfaction. Ultimately, those professionals with burnout regard themselves in a negative fashion, especially in their work with clients [5, 6].

The medical effect of burnout on healthcare practitioners, as well as on their personal lives, could be extremely serious. It could include the risk of myocardial infarction, coronary heart disease, reduced fibrinolytic capacity, reduced activity of the hypothalamic-pituitary-adrenal axis (HPA), and many other manifestations on an individual’s health [7].

In 1992, the World Health Organization’s (WHO) International Classification of Diseases (ICD-10) defined burnout as a “state of vital exhaustion” and as a problem related to “life-management difficulty” which could lead to depression. It was further defined in the revised ICD-11 with a more detailed definition [8].

### *Professional burnout in dentists*

Stressful long study years, financial burden, practice management problems, time-related pressure, difficult patients, heavy workloads, the routine aspect of the job, and many other stressors may increase the risk of burnout in dentists [9].

Lang also proposed that working in an isolated environment, confinement in a small workspace, constantly striving for perfection, time constraints, patient anxiety, and the dentist’s personality are all factors that contribute to burnout, in addition to the ergonomics of dentistry as a job. It is mentally and physically demanding, and as a result, muscle pain, back pain, circulatory disorders, and fatigue are common. Isolation due to long practice hours in a private practice setting and not having an opportunity to share and discuss problems with other colleagues may also be a factor which increases the burden. Compulsive attention to detail, extreme conscientiousness, control of emotions, and a marked dependence on individual performance and prestige are common personality traits in dentists which may lead to burnout. Lack of physical exercise can also contribute to mental and physical illness [10].

Lately, it was noticed that pediatric dentists in Egypt are not satisfied, after long years of studies, effort, and stress. They seem to be undergoing emotional exhaustion, depersonalization, and the feeling of lack of accomplishment. A pediatric dentist with symptoms of burnout will not perform as required specially with behavior management of children being an inevitable element in our job. Therefore, an affected pediatric dentist will definitely have a negative impact on the child and could lead to an unsuccessful treatment due to failure in behavior management. Here aroused the need of finding out to what extent this specific population is affected and to raise awareness in their community.

### *Aim*

The objectives of the present study were to determine prevalence of occupational burnout among Egyptian pediatric dentists and associated select demographic characteristics.

## Methods

This research was subject to an ethical committee review at the Faculty of Dentistry Ain Shams University and was approved (approval no. FDASU-Rec IM102003).

This study is a cross-sectional, observational study, which was carried by online survey questionnaire (Microsoft Forms) which was sent to active members in the Egyptian Pediatric Dentistry Associations, to faculty members at many of the Egyptian institutes through the current leaders.

A personalized introductory message reviewing informed consent along with an enclosed hyperlink to survey webpage was initially sent through WhatsApp followed afterwards by a reminder message sent 2 weeks later. All survey responses were anonymous without the use of any identification computer cookies, and respondent IP addresses were blocked for confidentiality. Participation was voluntary; survey respondents did not receive any remuneration for their participation in the study. The sample size calculations were based on the following assumptions: The minimum sample size will be 94 participants if the population size is 2500 and the confidence interval is 95% and marginal error of 5% and the proportion of response to the survey is 10%.

The sample size was calculated based on the following equation:

Sample size = $$\frac{\frac{z^2\times p\left(1-p\right)}{e^2}}{1+\frac{z^2\times p\left(1-p\right)}{e^2N}}$$

*N* = population size

*e* = margin of error

*z* = z score

A total number of 100 pediatric dentists participated in this study. All of them were offered to fulfill the questionnaires including that of Maslach Burnout Inventory and the semi-structured questionnaire to complete their sociodemographic and work-related data. Incomplete responses were excluded. The study was conducted from December 2020 to March 2021

MBI manual provides a table of norm scores (occupational subgroup—medicine) and cutoff points for categorization of each subscale score into high, moderate, and low level 4. Occupational burnout in the present study was defined as high scores in two dimensions: emotional exhaustion and depersonalization [11].

All the pediatric dentists were examined using the following tools:

1. A semi-structured sheet prepared by the researcher

Including sociodemographic data like gender, age, marital status, raising up their own children, and practicing in Egypt or not, number of years in practice, work hours per week, number of patients treated per day, the type of dental practice, level of education, enrolled in postgraduate studies or not, driving distance to work, and living with family or alone. The practitioners were also asked about the restriction of sedation and its impact on the job.

2) The Maslach Burnout Inventory

The Maslach Burnout Inventory (MBI) is the gold standard for measuring burnout. It consists of a 22-item questionnaire to evaluate the three independent dimensions of burnout: emotional exhaustion (range 0–54), depersonalization (range 0–30), and personal accomplishment (range 0–48).

where low (*EE* = 0–16, *DP* = 0–6, *PA* >= 39), moderate (*EE* = 17–26, *DP* = 7–12, *PA* = 32–38), and high scores (*EE* > = 27, *DP* > = 13, *PA* = 0–31) for each dimension are based on the low, medium, and high centiles of scores from a study of 1104 US doctors (Maslach and Jackson, 1986).

The data set was downloaded from Microsoft Forms and exported to Microsoft Excel and analyzed using Statistical Package for Social Sciences (SPSS) version 17.0 for data analyses.

Descriptive statistics (including frequency distribution analyses) and binary logistic regression were used with statistical significance set at P was coded, entered, and analyzed using SPSS (Statistical Package for Social Sciences) version 20. Descriptive statistics was done for study group as regards all collected variables. Univariate associations were tested using one-way ANOVA, Pearson’s correlation, chi-square, and Student *t*-tests.

Linear regression was used to calculate the standardized coefficients of regression and detect the significant variables and their effect on outcome (MBI subscales).

The probability of error (*P*-value) is used to indicate the level of significance:

3) *P* < 0.05: significant
4) *P* ≥ 0.05: nonsignificant

## Results

This study investigates the prevalence of the burnout syndrome at the level of its three dimensions among pediatric dentists in Egypt. Also, the researcher studied the relation between the sociodemographic data as well as work environment and the burnout syndrome. A total of 100 pediatric dentists were recruited.

Table 1 showed that 74% of our samples were females, 26% were males, most of the study sample aged between 20 and 40 years old, most of study samples were married and living with their family, and also, most of our participants have two or more kids (67.2%)

**Table 1:** Descriptive sociodemographic data

		*N*	%
Gender	Male	26	26.0%
	Female	74	74.0%
Age	20–30	22	22.0%
	30–40	61	61.0%
	40–50	12	12.0%
	50–60	2	2.0%
	Above 60	3	3.0%
Relationship status	Single	29	29.0%
	In a relationship	8	8.0%
	Married	56	56.0%
	Divorced	6	6.0%
	Widowed	1	1.0%
How many kids do you have (your own kids not patients)?	0	5	7.8%
	1	14	21.9%
	2	35	54.7%
	3	8	12.5%
	4 or more	2	3.1%
Are you living with your family?		91	91.0%

Table 2 showed more than 88% of the study group practice dentistry in Egypt, more than 55% have 5 to 15 years work experience, 63% treat 3 to 12 patient per day, 61% work from 10 to 40 h per week, 25% works more than 40 h per week, 55% work as group private practice, and 85% of the study group drive from 10 to 60 min to work and 86% live in city. Meanwhile, 92% of the study group think that unavailability of conscious sedation adds burden to their profession.

**Table 2:** Descriptive data about work environment

		*N*	%
Number of years in practice	0–5	22	22.0%
	5–10	31	31.0%
	10–15	27	27.0%
	15–20	10	10.0%
	20 or more	10	10.0%
Number of working hours per week	0–10	14	14.0%
	10–20	23	23.0%
	20–30	24	24.0%
	30–40	14	14.0%
	40 or more	25	25.0%
Average number of patients you treat per day	0–3	22	22.2%
	3–6	33	33.3%
	6–12	30	30.3%
	12 or more	14	14.1%
Governmental academic institution		40	40.0%
Private academic institution		17	17.0%
Governmental/public practice		22	22.0%
Group private practice		55	55.0%
Solo private practice		34	34.0%
Level of education	Resident	5	5.0%
	Certificate of pediatric dentistry (diploma/residency)	16	16.0%
	Master degree	50	50.0%
	Doctorate degree	18	18.0%
	Associate professor	4	4.0%
	Professor	7	7.0%
Are you currently enrolled in any further studies in pediatric dentistry?	Yes	50	50.0%
	No	50	50.0%
How long do you need to drive to work?	10–30 min	53	53.0%
	30–60 min	32	32.0%
	90 min	9	9.0%
	More than 90 min	6	6.0%
Where do you live?	City	86	86.0%
	Country side	14	14.0%
Do you think the unavailability of conscious sedation in practice adds burden to our profession?		92	92.0%

Table 3 shows that 62% of the study sample had high scores of emotional exhaustion, 21% of the study sample had moderate scores of emotional exhaustion, and 17% of the study sample had low scores of emotional exhaustion.

**Table 3:** Burnout dimensions among Egyptian pediatric dentists

	Low	Moderate	High
Exhaustion	17 (17%)	21 (21%)	62 (62%)
Depersonalization	43 (43%)	21 (21%)	36 (36%)
Personal accomplishments	23 (23%)	31 (31%)	46 (46%)

On the subscale of depersonalization, 43% of the study sample had low scores of depersonalization, 21% of the study sample had moderate scores of depersonalization, and 36% of the study sample had high scores of depersonalization,

On the subscale of personal accomplishment, 46% of the study sample had high scores of personal accomplishment, 31% of the study sample had moderate scores of personal accomplishment, and 23% of the study sample had low scores of personal accomplishment.

Table 4 shows that mean score of emotional exhaustion, depersonalization, and personal accomplishment was higher among male dentists, but the difference between both groups was not statistically significant (*P*-value > 0.05).

**Table 4:** Relation between burnout syndrome and socio-demographic variables

		Exhaustion	*t* (*p*-value)	Depersonalization	*t* (*p*-value)	Personal accomplishments	*t* (*p*-value)
		Mean ± SD		Mean ± SD		Mean ± SD	
Gender	Male (26)	31.81 ± 11.78	0.69 (0.492)	9.46 ± 5.83	1.28 (0.202)	34.38 ± 7.19	0.68 (0.501)
	Female (74)	29.92 ± 12.08		7.73 ± 5.94		33.23 ± 7.6	
Practice in Egypt	No (12)	26.67 ± 13.16	−1.16 (0.25)	7.33 ± 7.05	−0.53 (0.601)	35.25 ± 5.07	0.85 (0.398)
	Yes (88)	30.92 ± 11.79		8.3 ± 5.8		33.3 ± 7.74	
Governmental academic institution	No (60)	29.05 ± 12.09	−1.4 (0.165)	8.05 ± 6.29	−0.27 (0.790)	34.55 ± 7.14	1.69 (0.095)
	Yes (40)	32.45 ± 11.64		8.38 ± 5.42		32 ± 7.79	
Private academic institution	No (83)	30.12 ± 12.01	−0.53 (0.596)	8.28 ± 5.88	0.36 (0.720)	33.12 ± 7.58	−1.21 (0.228)
	Yes (17)	31.82 ± 12.02		7.71 ± 6.34		35.53 ± 6.78	
Governmental/public practice	No (78)	30.71 ± 12.59	0.46 (0.645)	8.24 ± 6.21	0.2 (0.841)	33.36 ± 7.77	−0.43 (0.669)
	Yes (22)	29.36 ± 9.63		7.95 ± 4.96		34.14 ± 6.47	
Group private practice	No (45)	32.31 ± 11.8	1.44 (0.152)	8.98 ± 6.1	1.22 (0.226)	33.27 ± 8.16	−0.32 (0.752)
	Yes (55)	28.85 ± 11.99		7.53 ± 5.76		33.75 ± 6.94	
Solo private practice	No (66)	29.48 ± 11.79	−1.08 (0.284)	7.67 ± 5.92	−1.21 (0.230)	33.03 ± 7.43	−0.93 (0.354)
	Yes (34)	32.21 ± 12.29		9.18 ± 5.92		34.5 ± 7.59	
Are you currently enrolled in any further studies in pediatric dentistry?	No (50)	29.4 ± 12.35	−0.84 (0.401)	7.82 ± 6.19	−0.6 (0.547)	35.08 ± 6.84	2.11 (0.038)
	Yes (50)	31.42 ± 11.62		8.54 ± 5.71		31.98 ± 7.83	
Where do you live?	City (86)	30.45 ± 12.38	0.09 (0.929)	7.99 ± 5.83	−0.8 (0.426)	33.53 ± 7.39	0.02 (0.987)
	Countryside (14)	30.14 ± 9.45		9.36 ± 6.65		33.5 ± 8.3	
Are you living with your family?	No (9)	29.11 ± 9.35	−0.34 (0.735)	6.89 ± 5.82	−0.68 (0.497)	33.11 ± 7.04	−0.18 (0.861)
	Yes (91)	30.54 ± 12.23		8.31 ± 5.96		33.57 ± 7.55	
Do you think the unavailability of conscious sedation in practice adds burden to our profession?	No (8)	31 ± 12.35	0.14 (0.885)	7.75 ± 7.01	−0.21 (0.832)	35.13 ± 6.1	0.63 (0.532)
	Yes (92)	30.36 ± 12		8.22 ± 5.87		33.39 ± 7.6	

Table 5 shows no statistically significant associations between age of dentists, number of years in practice and level of education, and the scores of the subscales of burnout. It was found that the number of kids you have is significantly associated with the level of depersonalization (*P*-value = 0.03).

**Table 5:** Correlation between subscales and sociodemographic data

	Exhaustion		Depersonalization		Personal accomplishments	
	Spearman correlation coefficient	*p*-value	Spearman correlation coefficient	*p* value	Spearman correlation coefficient	*p*-value
Age	0.005	0.963	−0.057	0.575	0.164	0.102
How many kids do you have (your own kids not patients)?	0.028	0.824	***0.271***	***0.030***	−0.024	0.851
Number of years in practice	0.025	0.802	0.036	0.725	0.180	0.073
Number of working hours per week	***0.199***	***0.047***	***0.306***	***0.002***	0.178	0.076
Average number of patients you treat per day	0.089	0.380	***0.205***	***0.042***	***0.455***	***< 0.001***
Level of education	0.039	0.703	0.033	0.747	0.009	0.927
How long do you need to drive to work?	0.159	0.114	0.131	0.195	−***0.198***	***0.048***

It was found that the number of working hours per week is significantly associated with the level of exhaustion and depersonalization.

It was found also that the average number of patients you treat per day is significantly associated with the level of depersonalization and personal accomplishment.

It was found also that how long do you need to drive to work is significantly associated with the level of personal accomplishment (*P*-value = 0.048).

Regression Analysis was also performed to have better understanding of the impact of each of the factors listed in our study. It was found that an increased number of patients treated per day by a pediatric dentist and the number of kids of his own he or she are raising could be strong perpetuating factors to Burnout (Tables 6 and 7).

**Table 6:** Regression analysis to identify perpetuating factors to burnout syndrome in the sample. Personal accomplishments. Are you currently enrolled in any further studies in pediatric dentistry? Average number of patients you treat per day. How long do you need to drive to work?

	B	*p*-value	95.0% confidence interval for B	
Average number of patients you treat per day	3.351	< 0.001	1.942	4.760
Are you currently enrolled in any further studies in pediatric dentistry?	−1.310	0.360	−4.137	1.517
How long do you need to drive to work?	−0.545	0.493	−2.116	1.026

*R* ^2^, 0.2

**Table 7:** Regression analysis to identify perpetuating factors to burnout syndrome in the sample. Depersonalization. How many kids do you have (your own kids not patients)? Number of working hours per week. Average number of patients you treat per day

	B	*p*-value	95.0% confidence interval for B	
Average number of patients you treat per day	0.878	0.307	−0.825	2.58
Number of working hours per week	0.473	0.463	−0.809	1.756
How many kids do you have (your own kids not patients)?	2.089	0.022	0.318	3.861

*R* ^2^, 0.16. Exhaustion, number of working hours per week. Only one factor was significant

## Discussion

Burnout in healthcare is on the rise. In 2022, the World Health Organization will embark on the development of evidence-based guidelines on mental well-being and will list burnout in the upcoming revision of the International Classification of Diseases as a syndrome conceptualized as resulting from chronic workplace stress that has not been successfully managed [12].

Maslach developed the burnout inventory which was named after him, which is easy to execute and yields reliable and valid information with which it is possible to evaluate and diagnose workplace burnout. Therefore, it was implemented in this study [11].

Emotional exhaustion, due to chronic exposure to unmitigated stress, makes clinicians feel emotionally, physically, and spiritually drained. This may result in affected individuals not feeling they can effectively give off themselves anymore. Often, they report being worn out, with loss of energy, depletion, debilitation, and fatigue. This may lead to depersonalization exhibiting inappropriate attitudes, sarcasm, and cynicism directed at others. Affected individuals may also experience irritability, loss of idealism, and withdrawal. Depersonalization and emotional exhaustion are closely associated, and the results may be a dental provider conveying the message that he or she does not care about the patient’s concerns or needs [9].

Compassion fatigue is associated with burnout because it expresses the fatigue and exhaustion a person can experience when dealing with difficult or unreasonable people. Compassion fatigue has been described as the convergence of secondary traumatic stress and cumulative burnout, a state of physical and mental exhaustion caused by a depleted ability to cope with one’s everyday environment [13]. A third aspect of burnout syndrome, reduced personal accomplishment, results in a tendency for people to demonstrate an inability to cope; they may have a negative impression of themselves and dissatisfaction with their work and accomplishments [4].

The present study surveyed the prevalence of occupational burnout (utilizing Maslach Burnout Inventory (MBI)) among pediatric dentists in the Egypt, and also, it aimed to study the sociodemographic characteristics of dentist suffering from burnout syndrome and to assess associated factors that increase the burnout syndrome and propose different recommendations to protect from it. A total of 74% of our sample were females, 26% were males, and most of the study sample aged between 20 and 40 years old similar to a study conducted in Saudi Arabia among postgraduate students in pediatric dentistry [14].

Most of study sample were married and living with their families; also, most of our participants had two or more kids (67.2%). More than 55% have 5 to 15 years work experience, 63% treats 3 to 12 patient per day, 61% works from 10 to 40 h per week, 25% works more than 40 h per week. 55% works as group private practice, 85% of the study group drives from 10 to 60 min to work, and 86% lives in the city.

At the outset of the study, it was hypothesized that pediatric dentists may be at greater risk for occupational burnout in comparison with other dentists because provision of pediatric dental care can be stressful with practitioners having to deal with anxious children and protective parents.

Regarding scores of MBI (prevalence of burnout syndrome), 62% of the study sample had high scores of emotional exhaustion, 36% of the study sample had high scores of depersonalization, 23% of the study sample had low scores of personal accomplishment, and the majority of subjects (97.2%) had high level of depersonalization and emotional exhaustion.

The level of personal accomplishment was low as 28%. In comparison with the US study, 23% has emotional exhaustion, 12% on the sub-scale of depersonalization, and 10% on personal accomplishment scale. This reveals that the burnout rates are higher in Egyptian pediatric dentists than US dentists. An estimate of 25% respondents fulfilled this study’s definition of occupational burnout (high emotional exhaustion + high depersonalization). This is also higher than dentists in the UK and similar to Irish dentists [15, 16]. This could be attributed to the fact that the study was conducted during covid-19 pandemic.

All the sociodemographic variables showed no significant effect on burnout scores except for distance from family, and being involved in further postgraduate studies exhibited extreme significance, revealing the stress levels to which a postgraduate candidate is exposed to in Egypt. It was found that being enrolled in a postgraduate pediatric dentistry program in Egypt is significantly related to high burnout rates. Most of the candidates work after their studying hours, to be able to meet their financial burden; therefore, this reflects significantly on the three subscales of burnout. This was similar to the study carried on postgraduate student in Saudi Arabia [14].

The present study found no gender differences regarding prevalence of burnout contrary to a random sample of US dentists wherein female pediatric dentists were more depressed than males. These results were contradicting to other studies reporting a significant association between female gender and emotional exhaustion [16–18]. There were no differences in prevalence of burnout in the present study based upon marital status, while married UK dentists had lower levels of depersonalization than single dentists. There was a higher prevalence of depression among US dentists who were single than those who were married. It was also reported by Gillespie et al. and Kumar whom are not in relationships may be at risk of high burnout levels as reported by other researchers [19, 20].

Moreover, working at a governmental academic institution seems to contribute to the burnout of a practitioner. It is not showing significance but stands on a borderline, as it could have been significant if the sample was larger.

There was no association between age of dentists, number of years in practice and level of education, and the scores of the subscales of burnout. While it was totally the opposite for the US pediatric dentists, as if experience exceeded 11 years in practice, the risk of having depersonalization is 2.99 times to candidates with less years in practice. It was found by Chohan et al. that working in group practice is better than running a single operatory [16].

On the other hand, it was found that the number of kids a pediatric dentist raises up is significantly associated with the level of depersonalization (*P*-value = 0.03). This may be due to more family duties are wanted after working hours, more stress on the dentists. It was also found that the number of working hours per week is significantly associated with the level of exhaustion and depersonalization. That was supported by Chohan et al. and Denton el al. confirming that those who worked 40 h or more per week were 10.59 times more likely to experience high emotional exhaustion in comparison to those working less than 20 h per week [16]. The study from Yemen found a significant association between the prevalence of burnout and working long hours [21]. Similar findings were reported by a study in some Arab countries which found healthcare professionals working for more than 40 h per week [22]. A lack of personal accomplishment and high emotional exhaustion were related to working long hours and a greater proportion of time spent working in NHS practice [15].

In the current study, it was revealed that long drives to work are also significantly associated with the low level of personal accomplishment (*P*-value = 0.048). Moreover, 92% of the study group believe that unavailability of conscious sedation adds burden to their profession. The use of sedation in dentistry has been prohibited in Egypt since 2008; moreover, even nitrous oxide gas use in dentistry has been completely banned. Reasons for the ban were not clear. Therefore, regulating the use of sedation for dental treatment will help in elevating some burden of the pediatric dentists shoulders and save the operating rooms precious time.

Findings from the present study however belied that notion over multiple MBI assessments. Mean emotional exhaustion and mean depersonalization scores of study respondents were significantly lower, while mean personal accomplishment scores were significantly higher than established MBI norms for overall population sample or medical professionals

Chohan et al. found a significant correlations in their study between moderate-to-severe depression and high emotional exhaustion, high depersonalization, and low personal accomplishment, where two out of five pediatric dentists with high MBI scores in both emotional exhaustion and depersonalization also had moderate-to-severe depression. These correlations confirmed other reports of the link between occupational burnout and depression in dentist, and the two conditions are discrete entities [16].

Given potential ramifications, raising awareness of mental health issues among pediatric dentists may be salutary even if benefit accrues only to a few. Individuals with depressive symptoms may have reluctance to seek treatment. In the USA, only 15% of the dentists suffering depression were receiving treatment; therefore, awareness is currently an essential need. It seems to be a worldwide problem which requires a bigger attention

### Limitations

1. We had a limited sample size, so there was underrepresentation for some categories in the sample like pediatric dentists having family burden, pediatric dentists having chronic illness, and pediatric dentists not satisfied with the specialty, so these variables were not correlated to burnout properly.
2. Some pediatric dentists refused to participate in the study; others were out of reach. We do not have sufficient data or information about those pediatric dentists specially whose rejection could be due to causes related to being actually suffering from burnout syndrome.
3. This is a cross-sectional study, so longitudinal studies and follow-up data may be needed in the future.

## Conclusions

- Burnout is a highly important issue to be taken care of in any profession dealing with people including medical professions and dentistry in particular and has its effects on nation’s productivity and service quality.
- Burnout prevalence among pediatric dentist in Egypt is high and higher than its prevalence among other dentists.
- Marriage and sleeping in home with the family have protective effects against burnout statistically.
- There is significant association between high number of working hours of the pediatric dentist per week and burnout.
- Awareness of such a condition is very important in the pediatric dentist’s community

## Data Availability

All data are available when needed.

## Abbreviations

HPA: Hypothalamic-pituitary-adrenal axis
WHO: World Health Organizations
ICD-10: International Classification of Disease-10
ICD-11: International Classification of Disease-11
MBI: Maslach Burnout Inventory
EE: Emotional exhaustion
DP: Depersonalization
PA: Personal accomplishment
NHS: National Health Services
US: United States
UK: United Kingdom
